# Development of beat-by-beat blood pressure monitoring device and nocturnal sec-surge detection algorithm

**DOI:** 10.1038/s41440-024-01631-9

**Published:** 2024-03-28

**Authors:** Ayako Kokubo, Mitsuo Kuwabara, Naoko Tomitani, Shingo Yamashita, Toshikazu Shiga, Kazuomi Kario

**Affiliations:** 1 Omron Healthcare Co., Ltd., Kyoto, Japan; 2 Division of Cardiovascular Medicine, Department of Medicine, Jichi Medical University School of Medicine, Tochigi, Japan

**Keywords:** Beat-by-beat blood pressure, Blood pressure surge in seconds, Blood pressure variability, Nocturnal blood pressure

## Abstract

The nocturnal blood pressure (BP) surge in seconds (sec-surge) is defined as a brief, acute transient BP elevation over several tens of seconds, triggered by obstructive sleep apnea (OSA) and sympathetic hyperactivity. Sec-surge imposes a significant strain on the cardiovascular system, potentially triggering cardiovascular events. Quantitative evaluation of sec-surge level could be valuable in assessing cardiovascular risks. To accurately measure the detailed sec-surge, including its shape as BP rises and falls, we developed a beat-by-beat (BbB) BP monitoring device using tonometry. In addition, we developed an automatic sec-surge detection algorithm to help identify sec-surge cases in the overnight BbB BP data. The device and algorithm successfully detected sec-surges in patients with OSA. Our results demonstrated that sec-surge was associated with left ventricular hypertrophy and arterial stiffness independently of nocturnal BP level or variability. Sec-surge would be worth monitoring for assessing cardiovascular risks, in addition to nocturnal BP level.

**Figure:**
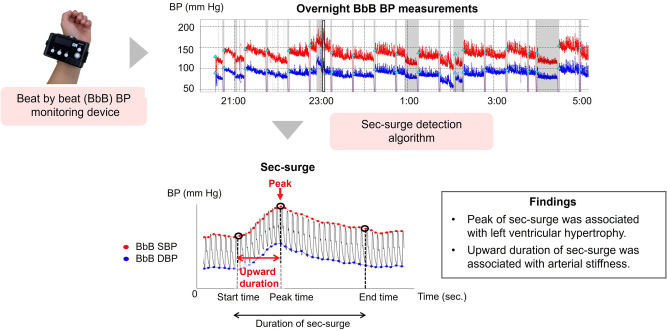
Nocturnal blood pressure (BP) surge in seconds (sec-surge) places heavy load on the cardiovascular system and can trigger cardiovascular events. To identify sec-surges, we developed a beat-by-beat BP monitoring device and a sec-surge detection algorithm. Furthermore, sec-surge was more related to cardiovascular risks than conventional nocturnal BP parameters.

Nocturnal blood pressure (BP) surge in seconds (sec-surge) places heavy load on the cardiovascular system and can trigger cardiovascular events. To identify sec-surges, we developed a beat-by-beat BP monitoring device and a sec-surge detection algorithm. Furthermore, sec-surge was more related to cardiovascular risks than conventional nocturnal BP parameters.

## Introduction

Managing nocturnal blood pressure (BP) is a crucial aspect in preventing cardiovascular disease [1–3]. Nocturnal BP obtained by ambulatory BP monitoring (ABPM) is a better predictor of cardiovascular events (CVEs) than daytime BP [4–9]. Recent studies have indicated that nocturnal BP, measured through home BP monitoring, also predicts future CVEs and correlates with target organ damage similar to ABPM [10–15]. Furthermore, nocturnal BP variability (BPV), defined as the standard deviation (SD) of nocturnal BP measurements obtained by ABPM, is independently associated with the risk of CVEs regardless of blood pressure level [16]. Nocturnal BPV is recognized to be associated with obstructive sleep apnea (OSA) [17, 18]. OSA and sympathetic nerve activity cause BP surge in seconds (sec-surge), which is characterized as an acute transient BP elevation over several tens of seconds [19–21]. Sec-surge can trigger CVEs and accelerate the progression of hypertensive target organ damage due to its intense pressure load. While ABPM is the gold standard for nocturnal BP monitoring, it fails to detect sec-surges due to its intermittent measurement nature.

We have developed an oxygen-triggered BP monitor that uses the cuff-oscillometric method to detect sec-surges. This monitor measures BP when oxygen saturation, continuously monitored by pulse oximetry, falls below a variable threshold [22–25]. We showed that the peak systolic BP (SBP) recorded by the oxygen-triggered BP monitor exceeds the mean value of nocturnal BP measured intermittently, similar to ABPM [23]. However, since this BP monitor uses the cuff-oscillometric method, it may not always detect the peak of the sec-surge. Continuous BP measurements is necessary for accurate detection of sec-surge.

To streamline sec-surge studies, we initially developed a continuous beat-by-beat (BbB) BP monitoring device using the tonometry method and evaluated its performance [26–28]. Subsequently, we developed an automatic sec-surge detection algorithm and evaluated its performance [29]. It is excessively time-consuming to manually identify all sec-surges occurring in a single night (>30,000 BbB BP recordings) across multiple patients, and no standard exists for sec-surge identification. We developed an algorithm to address these issues. Finaly, we demonstrated the clinical significance of sec-surge by assessing its association with cardiovascular disease risk, independent of conventional nocturnal BP variables [30, 31]. This article summarizes our latest findings.

## Development of a continuous, beat-by-beat blood pressure monitoring device

### Noninvasive approach for beat-by-beat blood pressure monitoring

We have considered three possible methods for noninvasive BbB BP monitoring, namely, applanation tonometry, volume clamp, and pulse transit time (PTT)-based BP estimation. The tonometry method directly measures BP using pressure sensors placed over a radial artery [32, 33]. In the volume clamp method, the cuff pressure equals the BP when the arterial volume, monitored by a photoplethysmograph, is kept constant through servo-controlled cuff pressure adjustments [34–36]. The PTT method calculates the BbB BP value by measuring the time delay of the pulse wave’s travel between two arterial sites. PTT is known to have an inverse relationship with BP [37, 38]. In recent years, cuffless BP measurement based on pulse wave analysis, including the PTT method, has been actively pursued [39].

Our goal is to implement daily BbB BP monitoring at home to achieve zero cerebro-cardiovascular events. The device must be compact, lightweight, and capable of measuring extended periods. In addition, direct pressure measurement may be preferable to the use of estimated BP values in the initial stages of BbB BP-related research. Tonometry method, which measures BP at lower levels than the commonly used oscillometric method in home BP monitors, are suitable for long-term use, such as overnight. The volume clamp is unsuitable for prolonged use as it restricts blood flow to the fingers. Furthermore, the device will be larger as it requires a pump, a valve, and an air hose for servo control. Despite the PTT method having the low user load for measurement, it only provides estimated BP values and is influenced by factors such as blood density and arterial compliance. The accuracy of the cuffless BP, including the PTT method, is currently under debate [40–42], and the 2021 European Society of Hypertension Guidelines on BP measurement does not recommend it for clinical use [43]. Therefore, we used the applanation tonometry method, which monitors actual pressure, to develop the BbB BP monitoring device.

### Development of a new blood pressure monitoring device based on tonometry

JENTOW-7700, a conventional BbB BP monitor based on tonometry from Nihon Colin, Japan, has received approval [44]. JENTOW, designed for use in medical facilities, is a large device that operates on alternating current power. We have developed a home BbB BP monitoring device based on tonometry [26, 31]; it is compact, lightweight, and rechargeable (i.e., no need to connect to a power supply when used).

**Fig. 1:**
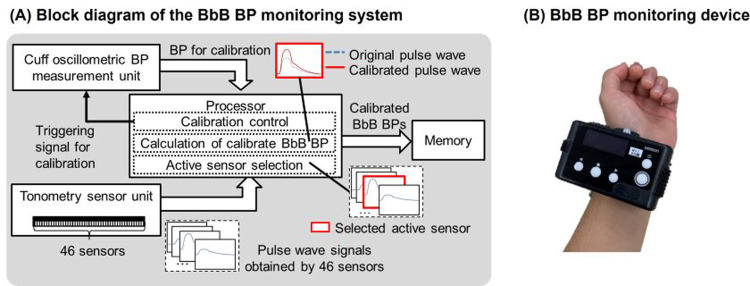
Overview of the BbB blood pressure monitoring system. **A** Block diagram of the BbB blood pressure monitoring system. **B** Tonometry sensor unit in the BbB BP monitoring device. BbB indicates for beat-by-beat. Source: Kokubo et al. [31]

Fig. 1A illustrates the block diagram of the BbB BP measurement system, and Fig. 1B depicts the device’s appearance. Pulse wave signals were recorded at 125 Hz from 46 pressure sensors, positioned over a radial artery on the skin. The amplitude of the pulse wave from 46 sensors was continuously monitored, with the sensor showing the highest amplitude selected as the active sensor. When the cuff-oscillometric BP measurement unit received the calibration trigger signal from the control function, it measured the calibration BP. The function automatically sent the triggering signal when the BP measurement began and significant changes occurred in the contact between the tonometry sensor and the skin due to body motion. The Calibrated BbB BP values were calculated from the pulse wave signal at the active sensor and the calibration BP value. The size of JENTOW was substantial due to its composition of a sensor unit and a control unit, connected by cables, with a total weight of 7000 g. Our BbB BP monitoring device seamlessly integrates a tonometry sensor, circuit board, and battery into a compact unit, achieving a lightweight design of 150 g. Patients can comfortably sleep overnight while wearing our rechargeable device, unbothered by cables.

**Fig. 2:**
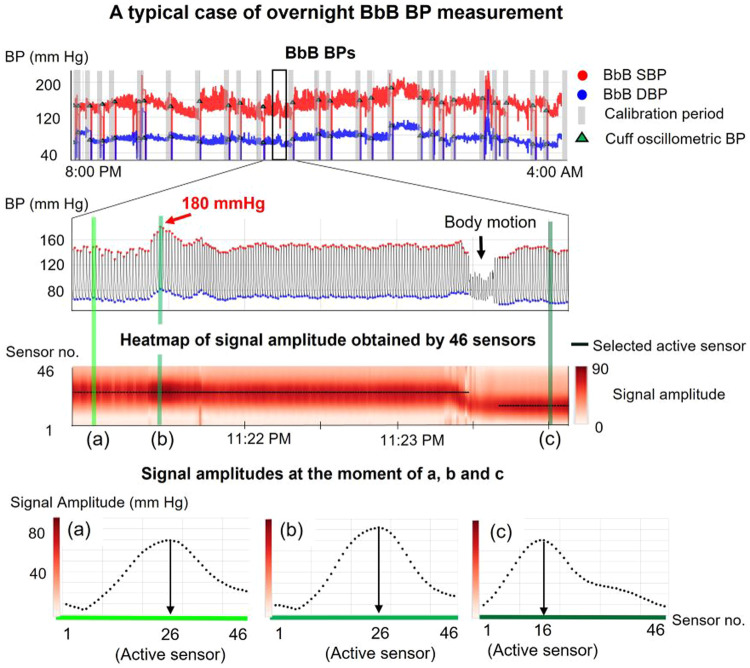
A typical case of overnight BbB BP monitoring. BbB BPs derived from the pulse wave signals at an active sensor. The active sensor is auto-selected based on the highest pulse wave signal amplitude among 46 sensors at any given moment. The heatmap represents the amplitude of pulse wave signals captured by 46 sensors over time, depicted as a colormap. (a)–(c) illustrate the amplitude of pulse signals from the 46 sensors just before the sec-surge, following the sec-surge, and after the active sensor is switched, respectively. BbB stands for beat-by-beat, BP for blood pressure, and sec-surge for blood pressure surge in seconds. Source: Kokubo et al. [31]

Figure 2 shows an example of overnight BbB BP monitoring. The heatmap indicates the amplitude of the pulse wave signals captured by 46 sensors, represented as a colormap. Moment (a) shows the signal amplitudes at the stable BP prior to the sec-surge. The (b) shows those at the peak of sec-surge. The (c) shows individuals with stable BP after switching the active sensor due to body movement. The active sensor was kept as 26 at both (a) and (b), even though the amplitude was increased due to the sec-surge. Despite switching the active sensor from 26 to 16 at point (c) due to body motion, the BP level remained nearly the same as at moment (a). Our device can measure BbB BP without calibration, provided the contact change between the skin and the tonometry sensor is slight.

### Evaluation of beat-by-beat blood pressure monitoring device

**Fig. 3:**
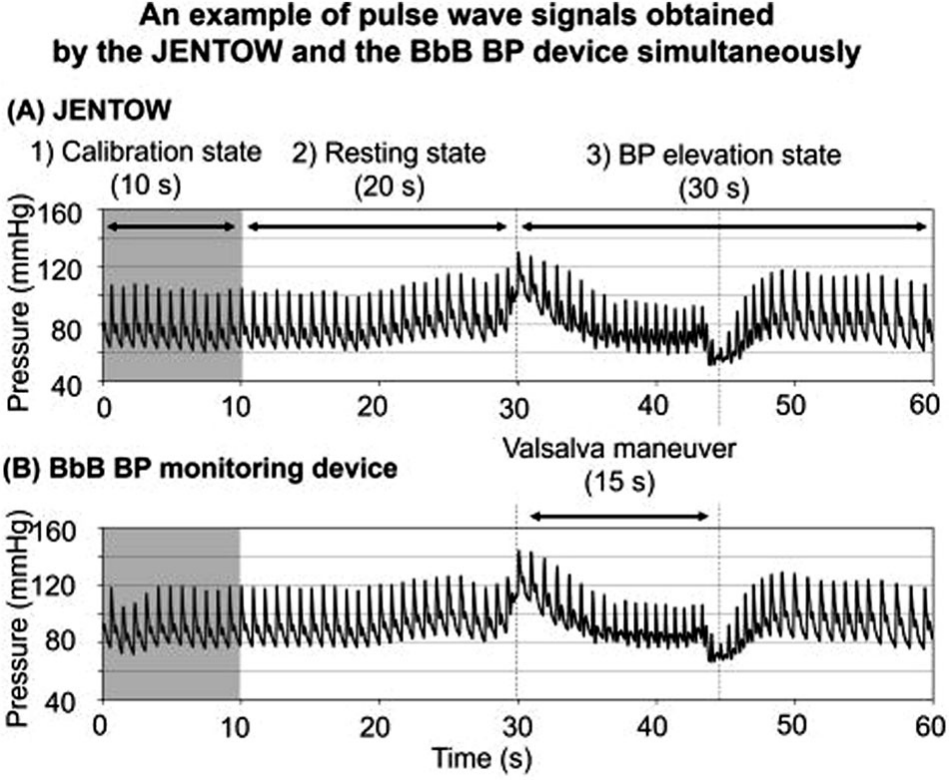
Example of the relationship between pulse wave signals simultaneously obtained by JENTOW on the right wrist (**A**) and the BbB BP monitoring device (**B**). BP indicates blood pressure, BbB device; beat-by-beat BP monitoring device. JENTOW is a conventionally validated BbB BP monitor that uses tonometry. Source: Ota et al. [26]. Reprinted with permission

The performance of the BbB BP monitoring device was evaluated in comparison to that of JENTOW. A total of 30 healthy subjects were recruited for this evaluation. The subject wore our device on their left wrist and JENTOW on their right, with BbB BPs measured simultaneously for 1 min. Each patient’s measurement was taken three times. Figure 3 shows an example of pulse wave signals, measured simultaneously, which include both resting and BP elevation states induced by the Valsalva maneuver. The performance was evaluated by the correlation coefficient of pulse wave signals from our device and JENTOW, as well as the difference in BbB BP values between the two devices (BbB device minus JENTOW). Consequently, 81 measurements were available, and a total of 4234 BbB BP values were obtained in the evaluation. The correlation coefficient between the pulse waves measured by the two devices was 0.98 ± 0.02 (mean ± SD) across 81 measurements [26]. The difference of BbB SBP between the two devices was −0.3 ± 4.7 mmHg, while the diastolic BP (DBP) difference was 0.7 ± 3.4 mmHg [26].

Our BbB BP monitoring device was significantly smaller and lighter compared to the approved BbB BP monitor (150 g vs. 7 kg). Furthermore, our device’s performance, evaluated under both stable and BP elevation state, was comparable to the approved BbB BP monitor. Our BbB BP monitoring device can accurately measure BP values in a clinical setting.

## Development of sec-surge detection algorithm

We have developed a sec-surge detection algorithm [29] for the efficient collection of sec-surge cases from clinical studies.

### Algorithm development

**Fig. 4:**
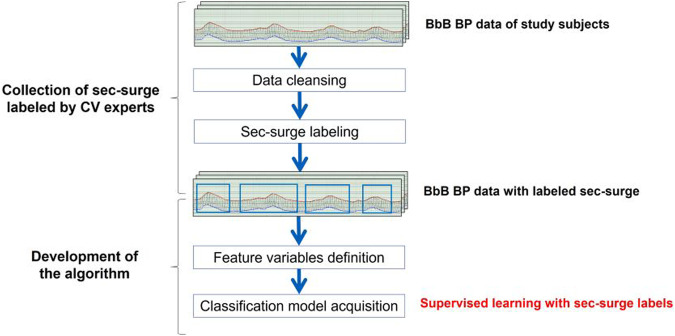
Overall framework for sec-surge detection algorithm development. Source: Kokubo et al. [29]

**Fig. 5:**
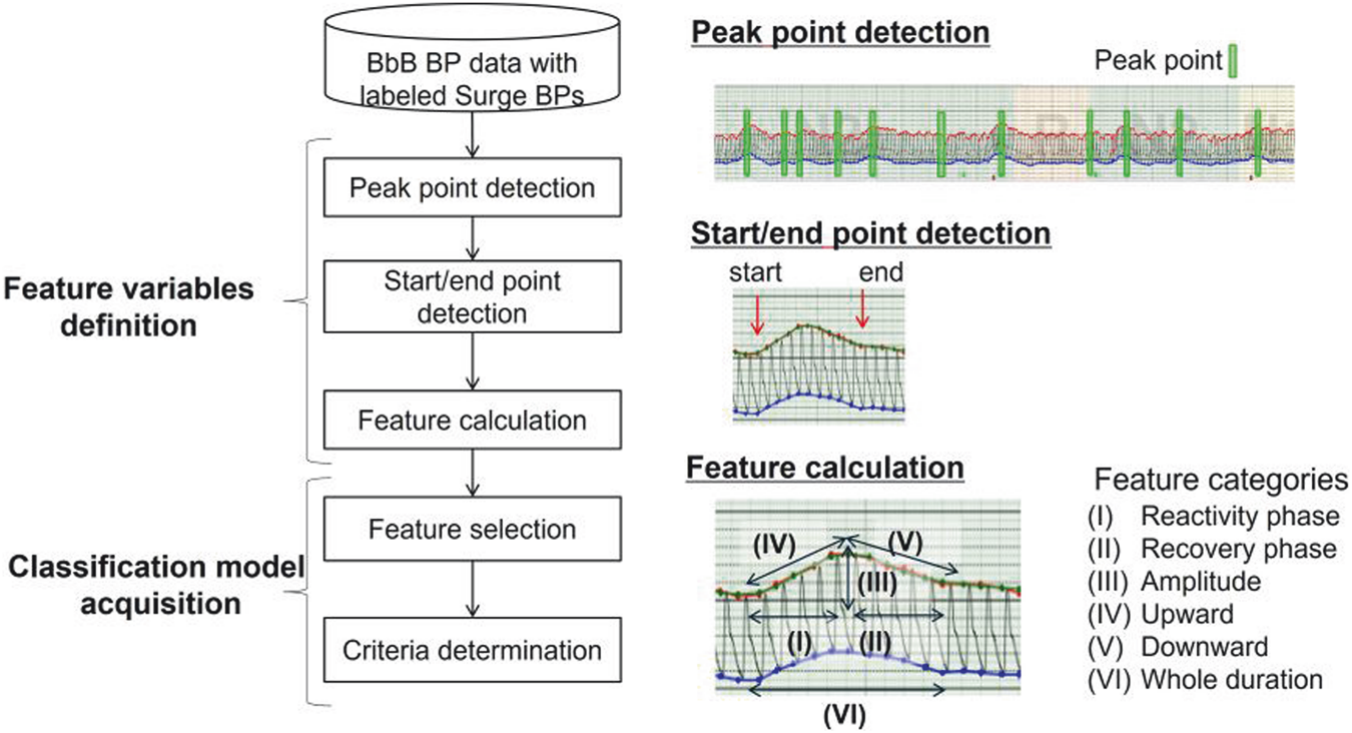
Steps in the development of the sec-surge detection algorithm. Source: Kokubo et al. [29]

The comprehensive development structure is depicted in Fig. 4. The algorithm was founded on supervised learning. A cardiovascular expert assessed a total of 3272 sec-surge labels from 94 nights of data in patients with suspected OSA. The sec-surge features were quantitatively defined using those labels, and a classification model was subsequently developed. The detailed steps for the algorithm’s development are shown in Fig. 5.

First, in the “feature variables definition” section, local maxima were identified from the BbB SBPs timeseries using a sliding-window. These were used as candidates for peak point of sec-surges. Second, the start point of sec-surge was identified prior to the peak, marking the end of stable BbB SBPs. The end point of sec-surge was identified as the point where the SBP decreased by 75% of the sec-surge amplitude (peak SBP–start SBP). Third, feature variables were calculated from the peak, start, end point, and the BbB SBPs between these points. A total of 48 sec-surge features were categorized into six groups: reactivity phase, recovery phase, amplitude, upward, downward, and whole duration, representing the clinician’s perspective on sec-surge. Through this procedure, sec-surge candidates were identified and subsequently classified as either sec-surge or NOT sec-surge in the subsequent “classification model acquisition” section.

The acquisition of a classification model comprises two parts: feature selection and the determination of criteria for each selected feature. In the feature selection, some features effective for classification were chosen from 48 features without duplicating the categories ensuring coverage of the clinician’s perspectives. The effectiveness of each sec-surge feature was evaluated using F-measure [45] by altering the threshold of the feature criterion. F-measure is a performance metric that harmonizes recall (sensitivity) and precision (positive predictive value). After the acquisition of effective features for classification, the criteria were determined established through a grid search. Each conditional expression was defined by whether the selected feature exceeded the set criteria (e.g., X ≥ A; where X represents the selected feature and A is X’s threshold). Candidates satisfying the AND operation of each conditional expression were identified as sec-surges.

### Algorithm evaluation

The performance was evaluated based on detection accuracy and average processing time, utilizing 3272 sec-surge labels in 94 overnight data sets. The algorithm’s detection performance was evaluated based on its correspondence with the sec-surge labels. The mean values of recall (defined as TP/(TP + FN)) and precision (defined as TP/(TP + FP)) were calculated using 5-fold cross-validation, where TP represents true positive, FN is false negative, and FP signifies false positive. The processing time to output the detection result for each overnight measurement data was measured and calculated the average value. The PC specifications for the measurement, commonly used in clinical practice, are as follows: The system had two Intel® Xenon® CPUs with a clock speed of 2.20 GHz each, and 7.5 GB of RAM.

**Fig. 6:**
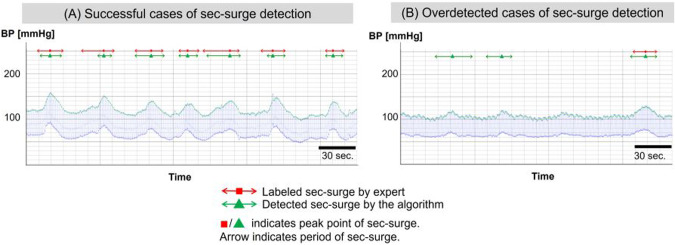
Typical instances of sec-surge detected by the algorithm. **A** Successful cases of sec-surge detection. **B** Overdetected cases of sec-surge detection. Source: Kokubo et al. [29]

Figure 6 shows typical detection cases as identified by the algorithm. The (A) depicts a patient experiencing numerous sec-surges within a brief period, each of which was successfully detected. The (B) depicts the cases of over-detection. Despite detecting three cases in the (B), two cases on the left side in the figure were not labeled. The recall of the detection was 0.90 ± 0.03 and the precision was 0.64 ± 0.05, using 5-fold cross-validation [29]. The algorithm’s average processing time for each set of overnight data was 11.0 ± 4.7 s [29].

If clinicians manually identify sec-surges from overnight BbB BP data, it will be time-consuming and the identification criteria will vary both within and between individuals. Given our algorithm’s ability to swiftly and sensitively detect sec-surges, it holds significant potential for clinical practice. Furthermore, the detection rule was clear and acceptable for physicians using this algorithm in their clinical practice.

## Sec-surge as a new clinical indicator

We assessed the clinical relevance of sec-surge by independently evaluating its association with left ventricular hypertrophy (LVH) and arterial stiffness, irrespective of conventional nocturnal BP variables [30, 31]. Additionally, we evaluated the difference of sec-surge severity between sleep apnea (SA)-related sec-surges induced by apnea/hypopnea or oxygen desaturation, and non-SA-related sec-surges induced by sympathetic nerve activity [31].

**Fig. 7:**
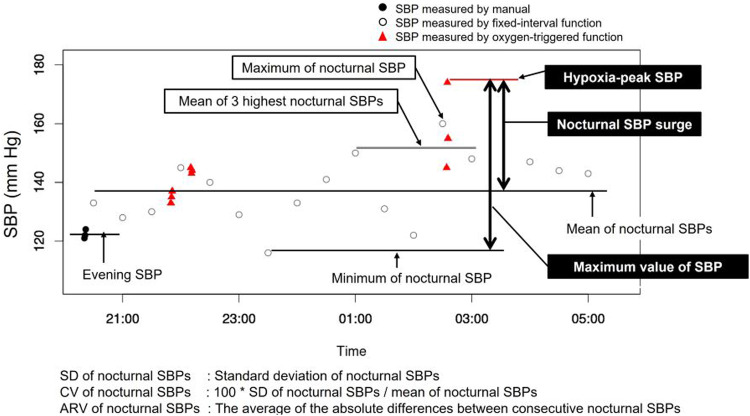
Definition of nocturnal blood pressure variables measured by cuff-oscillometric method. SBP stands for systolic blood pressure, SD for standard deviation, CV for coefficient of variation, and ARV for average real variability. Source: Kokubo et al. [31]

**Fig. 8:**
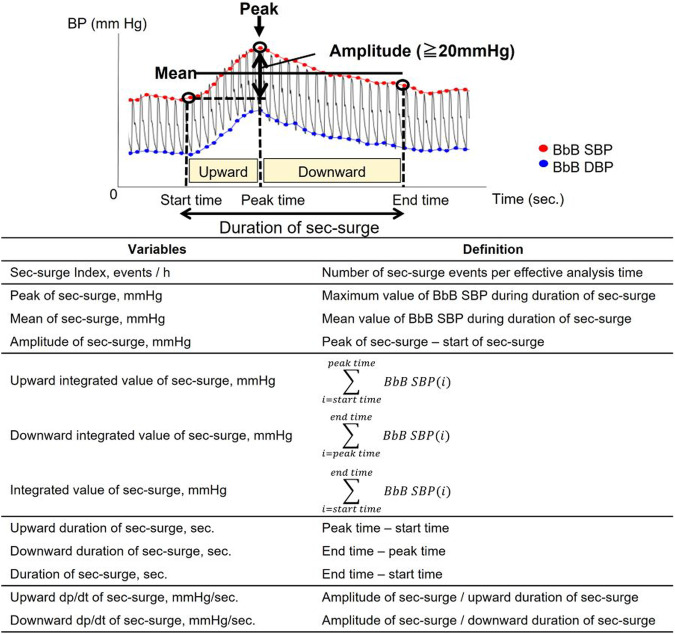
Definition of nocturnal blood pressure surge in seconds (sec-surge) variables. BbB stands for beat-by-beat, SBP for systolic blood pressure, and DBP for diastolic blood pressure. Source: Kokubo et al. [31]

A total of 41 patients, suspected of having OSA and diagnosed with nocturnal hypertension, were included in the evaluation [30, 31]. The patients underwent overnight full polysomnography (PSG) and BP measurements using the BbB BP monitor on their left wrist and a cuff-oscillometric BP monitor on their right arm. All patients were included in the analysis of the sec-surge severity between SA-related and non-SA-related sec-surges observed in the same overnight measurement data. To analyze the association with LVH, we included 18 patients with LV mass data measured by cardiac MRI in an outpatient setting. Similarly, for the association with arterial stiffness, we included 34 patients with outpatient-measured pulse wave data. We defined the conventional nocturnal BP variables, as illustrated in Fig. 7, and the sec-surge variables, as illustrated in Fig. 8 [31]. We described the details of these variables in our previous study. The peak, start, and end points of sec-surges were detected by the above-mentioned algorithm.

### Severity comparison between SA-related and non-SA-related sec-surges

The severity of the sec-surge was characterized by its peak, amplitude, and the number of sec-surges [31]. The peak of sec-surge was defined as the highest value of BbB SBPs during sec-surge, while the amplitude of sec-surge was determined by subtracting the starting SBP from the peak SBP. The PSG software analysis results were used to categorize all sec-surges into SA-related and non-SA-related sec-surges, taking into account the incidence of apnea/hypopnea and oxygen desaturation.

No significant differences were observed in the severity of sec-surges between SA-related and non-SA-related sec-surges (number of sec-surges: 19.5 ± 26.0 vs. 16.4 ± 29.8 events, peak: 148.2 ± 18.5 vs. 149.3 ± 19.2 mmHg, amplitude: 26.0 ± 4.3 vs. 25.8 ± 5.7 mmHg, arranged according to SA factor and non-SA factor) [31]. Our findings align with prior research, which found no significant differences between SA-related and non-SA-related BP elevation as assessed by the PTT method [46].

Previous studies [46, 47] have evaluated SA-related BPV using the PTT method or pulse decomposition analysis (PDA) in patients with OSA or suspected OSA. Their SA-related BPV, nearly equal to sec-surge, was smaller than in our study. The peak of sec-surge was 148 mmHg (tonometry) vs. 127 ~ 130 mmHg (PTT) vs. Not Applicable (PDA). The amplitude of sec-surge was 26 mmHg (tonometry) vs. 4 ~ 7 mmHg (PTT) vs. 14 mmHg (PDA). The tonometry method directly measures pressure via sensors, and the sec-surge detection algorithm provides detailed evaluations of each sec-surge case. Consequently, our study assessed sec-surges more accurately compared to previous studies.

### Association with left ventricular hypertrophy and arterial stiffness

We assessed left ventricular hypertrophy (LVH) using the left ventricular mass index (LVMI) and arterial stiffness using the brachial-ankle pulse wave velocity (baPWV). The relationship between these indices and BP variables was assessed using simple Pearson’s correlation and multiple regression analysis.

In the LVH analysis, a strong association was observed between the peak of sec-surge and LVMI (*r* = 0.607, *p* < 0.01, *n* = 18) [31]. Moreover, the association with LVMI persisted even when sec-surges were categorized into SA-related and non-SA-related sec-surges (SA factor: *r* = 0.551, *p* = 0.041, *n* = 14; non-SA factor: *r* = 0.606, *p* = 0.017, *n* = 15). The multiple regression analysis of LVMI, using two independent variables (sec-surge and conventional BP), revealed significant associations between the peak of sec-surge and LVMI, independent of conventional nocturnal BP level and variability.

In the analysis of arterial stiffness, the upward duration of sec-surge, defined as the time from start to peak point, was significantly associated with baPWV (*r* = 0.481, *p* < 0.01, *n* = 34) [30]. Similar to the LVH analysis, the association with baPWV persisted even when sec-surges were categorized into SA-related and non-SA-related sec-surges (SA factor: *r* = 0.419, *p* = 0.024, *n* = 29; non-SA factor: *r* = 0.473, *p* < 0.01, *n* = 30). In the multiple regression analysis of baPWV, adjusted for age and sex, the upward duration of sec-surge was independently associated with baPWV, irrespective of conventional nocturnal BPV, but not with nocturnal BP level.

The studies assessing the correlation between nocturnal BP parameters and LVMI or PWV are consolidated in Tables 1 and 2. Studies referenced in Table 1 indicate that nocturnal BP level independently associates with LVMI [48, 49], whereas nocturnal BPV does not [50, 51]. Similarly, Table 2’s related studies indicate that nocturnal BP level independently associates with PWV [49, 52–54]. However, nocturnal BPV’s association with PWV was less significant than nocturnal BP level, albeit independent [53, 55]. The correlation strength for conventional nocturnal BP parameters in our results [30, 31] consistently aligned with these pieces of evidence. Furthermore, the sec-surge had a stronger correlation with LVMI and baPWV than conventional nocturnal BP parameters in our simple correlation analysis. Its association with LVMI was also independent of the nocturnal BP level. Sec-surge was not significantly associated with baPWV independently of nocturnal BP level, but it was significant independently of nocturnal BPV. In assessing cardiovascular risks related to LVH and arterial stiffness, it may be beneficial to monitor sec-surge alongside nocturnal BP levels.

**Table 1:** Association between nocturnal BP parameters and LVMI in related studies

Author (year)	Study subjects	Cardiovascular parameter	Nocturnal BP parameter (measurement length or timing)	Simple correlation	Significance after adjusting for BP level	Adjusted covariates in multivariate analysis
Schillaci et al. (1998) [50]	Untreated hypertensive patients (*n* = 1822)	LVMI (echocardiography)	SD of nocturnal SBP (ABPM)	*r* = 0.1, *p* < 0.01	No	Age, height, 24-h SBP, 24-h DBP, BMI, duration of hypertension, alcohol intake, the amount of daily cigarette smoking (the model was separately analyzed in each sex)
Bilo et al. (2007) [51]	Patients with hypertension, either untreated or under stable antihypertensive treatment, for at least 1 month. (*n* = 339)	LVMI (echocardiography)	SD of nocturnal SBP (ABPM)	*r* = 0.04, NS	N/A	N/A
Cuspidi et al. (2013) [48]	Population-based subjects (*n* = 1682)	LVMI (echocardiography)	Mean of nocturnal SBP (ABPM)	*r* = 0.365, *p* < 0.01	Yes	Age, sex, weight, office SBP, use of antihypertensive drugs (identified with stepwise selection)
Kario et al. (2015) [49]	Patients with one or more cardiovascular disease risk (*n* = 2563)	LVMI (echocardiography)	Mean of nocturnal SBP (2,3,4 a.m. HBPM)	*r* = 0.18, *p* < 0.001	Yes	Age, sex, BMI, use of antihypertensive drug, evening or bedtime dosing of antihypertensive drug, sleep duration, office BP
Kokubo et al. (2021) [31]	Patients with nocturnal hypertension and suspected OSA (*n* = 18)	LVMI (cardiac MRI)	Peak of Sec-surge (Overnight BbB BP)	*r* = 0.607, *p* < 0.01	(1) Yes (2) Yes	(1) Nocturnal SBP level (2) Nocturnal BPV
			Mean of SBP (Overnight every 30 min)	*r* = 0.240, *p* = 0.338	N/A	N/A
			SD of SBP (Overnight Every 30 min)	*r* = −0.066, *p* = 0.794	N/A	N/A

*ABPM* ambulatory blood pressure monitoring, *BbB* beat-by-beat, *BMI* body mass index, *BP* blood pressure, *BPV* blood pressure variability, *DBP* diastolic blood pressure, *HBPM* home blood pressure monitoring, *LVMI* left ventricular mass index, *OSA* obstructive sleep apnea, *SBP* systolic blood pressure, *SD* standard deviation, *sec-surge* blood pressure surge in seconds

**Table 2:** Association between nocturnal BP parameters and PWV in related studies

Author (year)	Study subjects	Cardiovascular parameter	Nocturnal BP parameter (measurement length or timing)	Simple correlation	Significance after adjusting for BP level	Adjusted covariates in multivariate analysis
Syrseloudis et al. (2011) [52]	Untreated hypertensive patients (*n* = 119)	log (cfPWV)	Mean of nocturnal SBP (ABPM)	*r* = 0.37, *p* < 0.001	N/A (Although 24-h SBP was not included in the regression model, the standardized β of nocturnal SBP in the model was stronger than that of 24-h SBP in another regression model adjusted for the same covariates)	Age, sex, waist circumference, 24-h heart rate, log (hs-CRP), uric acid
Schillaci et al. (2012) [55]	Untreated hypertensive patients (*n* = 2089)	cfPWV	SD of nocturnal SBP (ABPM)	*r* = 0.18, *p* < 0.01	Yes (but partial correlation coefficient in 24-h SBP was stronger than nocturnal SD)	Age, sex, 24-h SBP
Garcia et al. (2013) [53]	Hypertensive patients (*n* = 344)	cfPWV	Mean of nocturnal SBP (ABPM)	N/A	Yes	Age, sex, 24-h SBP, 24-h heart rate, antihypertensive drugs
			SD of nocturnal SBP (ABPM)	*r* = 0.199, *p* < 0.001	N/A	N/A
Kario et al. (2015) [49]	Patients with one or more cardiovascular disease risk (*n* = 2563)	baPWV	Mean of nocturnal SBP (2,3,4 a.m. HBPM)	*r* = 0.31, *p* < 0.001	Yes	Age, sex, BMI, use of antihypertensive drug, evening or bedtime dosing of antihypertensive drug, sleep duration, office BP, morning SBP, evening SBP
Liu et al. (2022) [54]	Untreated non-dipper hypertensive patients (*n* = 77)	baPWV	Mean of nocturnal SBP (ABPM)	*r* = 0.297, *p* < 0.01	Yes	Age, sex, weight, height, BMI, 24-h SBP, night-to-day SBP ratio
Kokubo et al. (2022) [30]	Patients with nocturnal hypertension and suspected OSA (*n* = 34)	baPWV	Upward duration of sec-surge (Overnight BbB BP)	*r* = 0.481, *p* < 0.01	(1) No (2) Yes	(1) Age, sex, nocturnal SBP level (2) Age, sex, nocturnal BPV
			Mean of nocturnal SBP (Every 30 min)	*r* = 0.343, *p* = 0.058	N/A	N/A
			SD of nocturnal SBP (Every 30 min)	*r* = 0.070, *p* = 0.698	N/A	N/A

*ABPM* ambulatory blood pressure monitoring, *baPWV* brachial-ankle pulse wave velocity, *BbB* beat-by-beat, *BMI* body mass index, *BP* blood pressure, *BPV* blood pressure variability, *cfPWV* carotid-femoral pulse wave velocity, *DBP* diastolic blood pressure, *HBPM* home blood pressure monitoring, *LVMI* left ventricular mass index, *OSA* obstructive sleep apnea, *SBP* systolic blood pressure, *SD* standard deviation, *sec-surge* blood pressure surge in seconds

In conclusion, the peak of sec-surge was significantly associated with LVH independently of conventional nocturnal BP level, while the upward duration of sec-surge was associated with arterial stiffness independently of conventional nocturnal BPV. Furthermore, even when sec-surges were categorized based on the triggering factors (SA/non-SA), the associations maintained the same level for both LVH and arterial stiffness. This implies that sec-surge can assess both cardiac and vascular loads, regardless of the triggering factor.

## Challenges of emerging technology

Sec-surges were successfully detected using the tonometry-based BbB BP monitoring device and sec-surge detection algorithm. Moreover, sec-surge can be beneficial in assessing cardiovascular load as well as nocturnal BP level. Nonetheless, some issues remain. First, tonometry-based measurements were sensitive to wrist movement. Maintaining an extended and supinated wrist position facilitates arterial pulsation detection for the tonometry method. In other words, its durability decreases during flexion and pronation. The device requires an enhanced mechanical structure to withstand flexion and pronation, or a fixing tool between the device and the patient’s wrist. Although recalibration occurring when significant BP changes were induced by movement, the optimal frequency and timing of calibration, which ensures measurement reliability without disrupting the patient’s sleep, should be considered. Second, general users often find it challenging to detect the radial artery position by palpation. Although the BbB BP monitoring device having a function to adjust the sensor position within a limited range, there is a need to enhance the position guidance for non-medical personnel. Third, the BbB BP monitoring device did not have a height-correction function according to changes in the body or arm position. In addition, if the blood flow in the radial artery is inhibited by sleeping, such as when the arm is placed under the body, the BP value will be affected. This issue is not unique to our device but is also noted in other nighttime BP monitors, particularly those that measure wrist BP. Fourth, the performance evaluation indicated that the sec-surge detection algorithm tended to over-detect cases. Most overdetected cases were subtle BPV and barely met the sec-surge criteria. The labeling staff, despite over a week’s training, found it challenging to identify them as sec-surge. However, since the algorithm partially detected the noisy BPV, it is necessary to manually check the detection results and eliminate any evident noisy BPV during its use. We executed this procedure before the quantitative analysis of sec-surge, adhering to the sec-surge classification conditions (sec-surge/ undetermined BPV/ noisy BPV) outlined in our prior study [29]. Fifth, the algorithm was not applicable to patients with atrial fibrillation who have large variations in BbB BPs.

## Conclusion and perspectives

To identify sec-surges that can trigger CVD events and strain the cardiovascular system, we developed the BbB BP monitoring device and the sec-surge detection algorithm. The sec-surge detection algorithm can significantly expedite the identification of sec-surge cases in overnight BbB BP data. The developed BbB BP monitoring device, compact and lightweight, demonstrated that performance was comparable to the conventional, tonometry-based, validated continuous BP monitor. Nonetheless, certain issues such as measurement robustness, calibration frequency, and usability still persist. In a future study, we will enhance the device and conduct validation. Moreover, our findings indicated that, besides nocturnal BP level, the peak and upward duration of sec-surge were significant in assessing LVH and arterial stiffness. While this study partially demonstrated the relationship between sec-surge and cardiovascular risks, further prospective studies are required for a comprehensive evaluation.
